# Notch-associated lncRNAs profiling circuiting epigenetic modification in colorectal cancer

**DOI:** 10.1186/s12935-022-02736-2

**Published:** 2022-10-13

**Authors:** Omnia Emam, Eman F. Wasfey, Nadia M. Hamdy

**Affiliations:** 1 Egyptian Drug Authority, Cairo, Egypt; 2 Biochemistry Department, Faculty of Pharmacy, Ain Shams University, Cairo, 11566 Egypt

**Keywords:** Epigenetics, lncRNAs, Notch, Colorectal cancer, Inflammation, Micro-RNAs, Hallmarks of cancer

## Abstract

**Background:**

Colorectal cancer (CRC) is one of the most prevalent digestive cancers, ranking the 2nd cause of cancer-related fatality worldwide. The worldwide burden of CRC is predicted to rise by 60% by 2030. Environmental factors drive, first, inflammation and hence, cancer incidence increase.

**Main:**

The Notch-signaling system is an evolutionarily conserved cascade, has role in the biological normal developmental processes as well as malignancies. Long non-coding RNAs (LncRNAs) have become major contributors in the advancement of cancer by serving as signal pathways regulators. They can control gene expression through post-translational changes, interactions with micro-RNAs or down-stream effector proteins. Recent emerging evidence has emphasized the role of lncRNAs in controlling Notch-signaling activity, regulating development of several cancers including CRC.

**Conclusion:**

Notch-associated lncRNAs might be useful prognostic biomarkers or promising potential therapeutic targets for CRC treatment.

Therefore, here-in we will focus on the role of “Notch-associated lncRNAs in CRC” highlighting “the impact of Notch-associated lncRNAs as player for cancer induction and/or progression.”

**Graphical Abstract:**

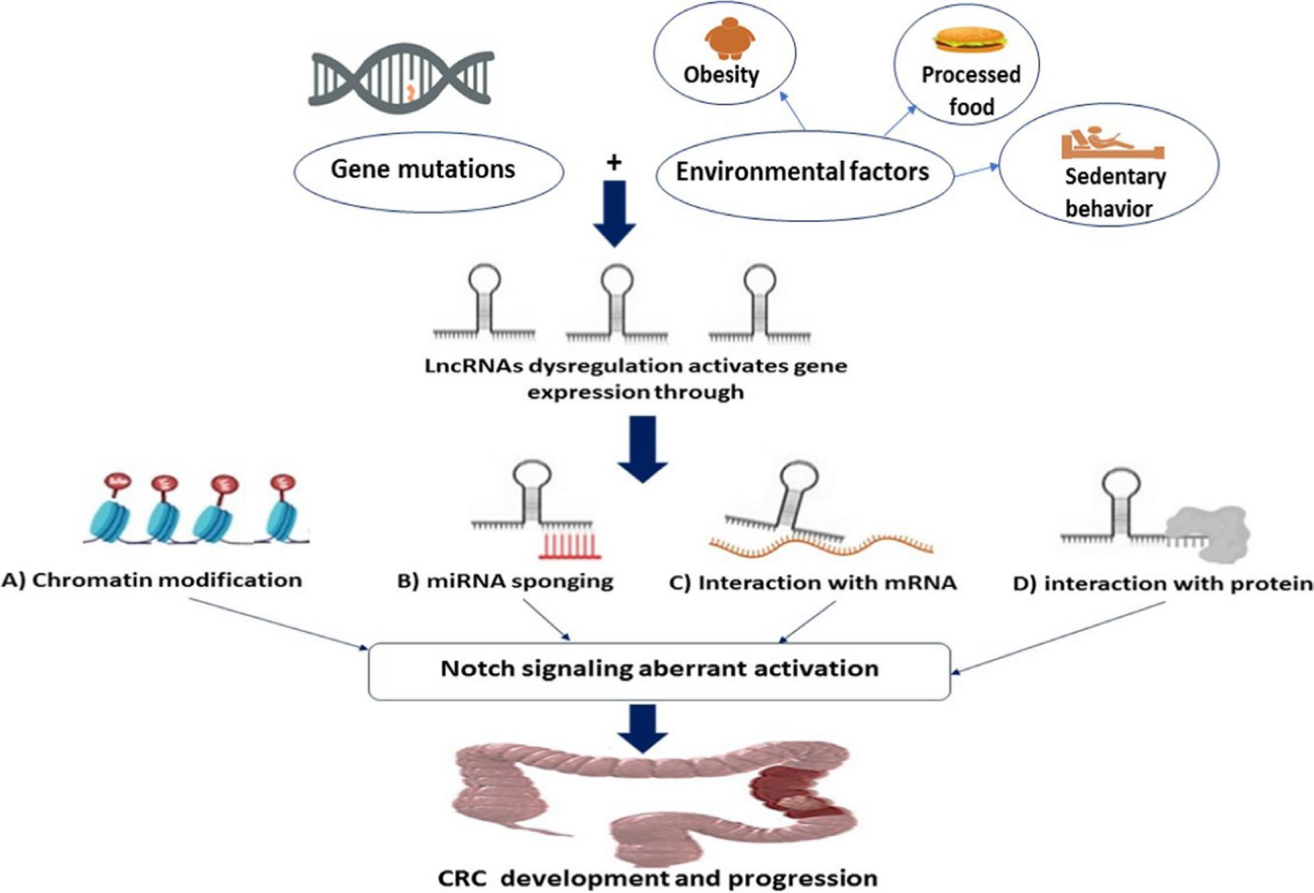

## Introduction

### Background

CRC is the third most ubiquitous malignancy as well as the second major cause of cancer-correlated death worldwide [1]. Surprisingly, CRC is now becoming more common among adolescents and young adults under the age of 45, who frequently come with advanced disease [2]. the prevalence of CRC is globally estimated to grow, environmental factors, such as increased sedentary behavior and obesity or processed food are thought to be one of the driving risk factors to this increase [3]. Even after surgical resection, chemotherapy/radiotherapy, using immunotherapy or the state-of-the-art targeted therapy, but, unfortunately, the 5-year survival rate remains low [2]. Cancer recurrence and distant metastases are the cause for these poor outcomes, especially for more advanced stage CRC [4].

CRC develops, throughout time, like other cancer types, as a result of a cascade of *epigenetic alterations*, mostly, affecting the genetic component, driving the normal colonic mucosa conversion into malignant tumor [5]. This interconversion is generated from alterations occurring within polyps, mostly adenomas [6]. Recent evidence shows that aberrant Notch cascade is crucial for CRC evolution.

Notch gene was discovered in 1917 by Morgan et al. in mutant Drosophila. The gene is known as 'Notch' because it causes a “breach” in the wings of Drosophila [7]. Notch cascade is conserved, among species, to control variety of biological activities as cell proliferation, differentiation as well as regulating cell fate decisions [8, 9].

Deregulation of Notch pathway is related to the development of hematological and solid malignancies, via pro-tumorigenic effect [10–13]. In the intestine, Notch pathway controls the homeostatic self-renewal processes and can cause ulcerative colitis, if the Notch pathway was dysregulated [14], which would cause the tumorigenic transformation of epithelia [15].

Notch pathway is a key player in CRC from initiation to resistance and metastasis, driving CRC progression and/or poor overall survival (OS) [16–19]. Positive association has been shown between the Notch receptor, Notch1, expression and deeper invasion of tumor-lymph node-metastasis (TNM) in CRC [20]. Patients with Notch1-positive malignancies had a worse OS rate than those with Notch1-negative ones [20]. Moreover, Notch-signaling is an ultimate regulator of epithelial-mesenchymal transition (EMT) process [21]. Notch-induced-EMT is a fundamental factor in CRC stemness and aggressiveness [22]. Also, increased expression of Notch as well as its target genes was shown to contribute to CRC chemoresistance [23–26].

Epigenetics-influenced activation of the Notch pathway would be led by non-protein coding RNAs (ncRNAs) expression dysregulation [27, 28]. LncRNAs are non-protein producing transcripts, performing a crucial role in the epigenetic regulation(s) affecting gene expression [29]. LncRNAs can control Notch-activation through regulation of Notch receptors or Notch ligands expression, either on transcriptional or post-transcriptional levels [30]. On the other hand, some lncRNAs are Notch-signaling downstream targets [31]. Several studies have showed that dysregulated lncRNAs have implications in CRC development, progression, metastasis as well as developing chemoresistance affecting the disease clinical outcomes [32–34].

Therefore, the interest in this review is to focus on the “Impact of the Notch-associated lncRNAs in CRC”. The review first aims to briefly discuss lncRNAs', Notch-signaling pathway and Notch-associated-lncRNAs mechanism(s) profiling in cancer. LncRNAs interacting with the Notch-cascade contributing to the development of various tumors are presented in the review. Second, we will highlight the role of Notch-associated lncRNAs as a player in cancer induction and progression, after defining specifically CRC types. Moreover, describing “Notch-associated lncRNAs impact on CRC clinical outcomes” and the “Notch-associated lncRNAs relationship to multidrug resistance (MDR), metastasis or recurrence.”

### Non-protein coding RNA

Non-protein coding (non-coding) regions of the genome, generates numerous families of ncRNAs [35, 36], that controls gene expression and function. ncRNAs are classified based on their length, location and function into micro-RNAs (miRNAs), lncRNAs, small nucleolar RNAs (snoRNAs), small nuclear RNAs (snRNAs), small interfering RNAs (siRNAs) and PIWI-interacting RNAs (piRNAs) [37–39].

### Long non-coding RNA

LncRNA are molecules with a length of more than 200 nucleotides [40]. LncRBase; The lncRNA sequence database; LncRBase is freely available at http://www.lncRbase.org

LncRNAs were originally described in mice through large-scale sequencing of entire cDNA libraries [41]**.**

**Fig. 1:**
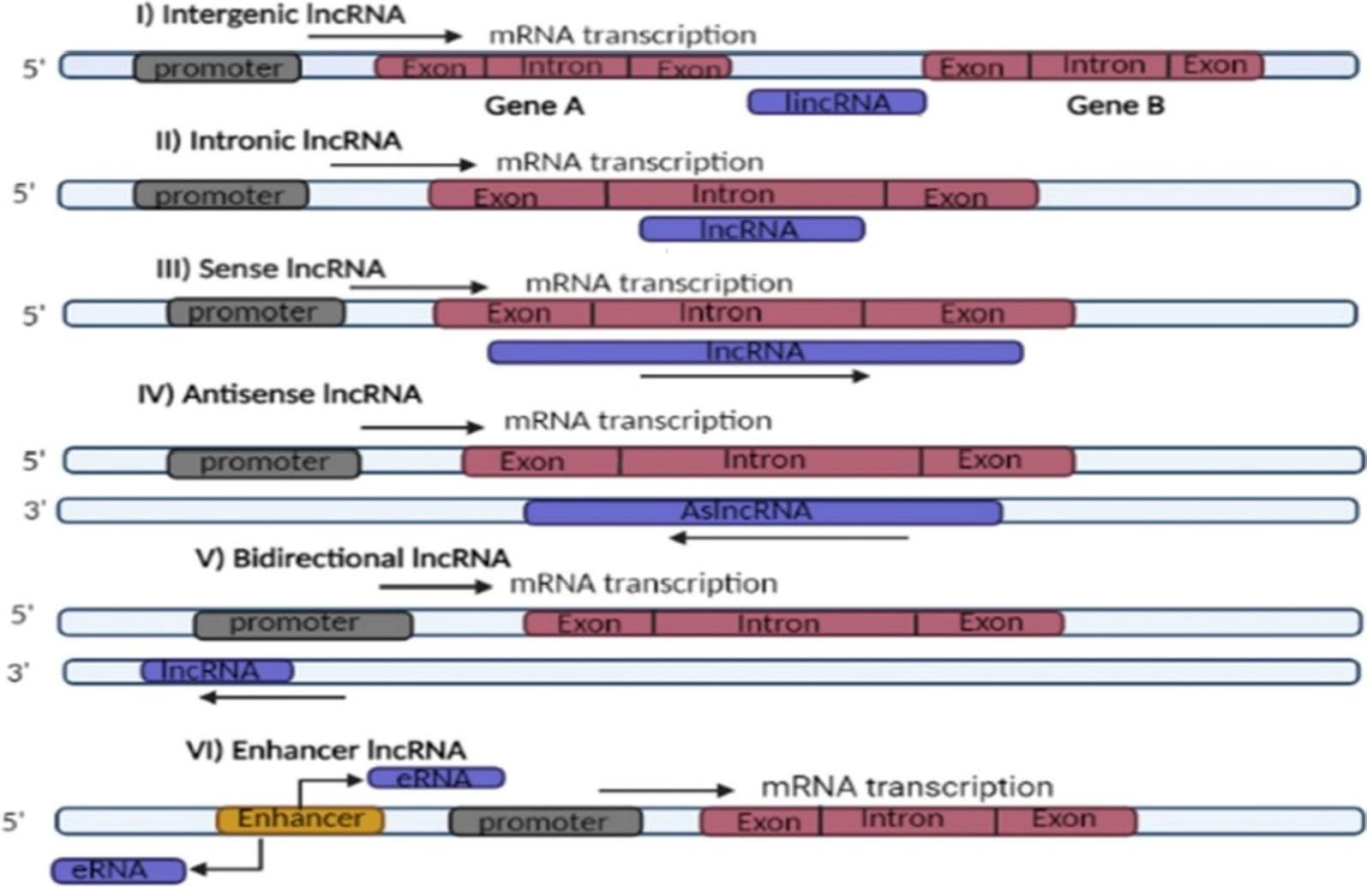
LncRNAs' classification based on their biogenesis site in relation to the coding genes. Depending on the biogenesis location, lncRNAs are classified into, intergenic which is transcribed from gaps between genes. Intronic which is transcribed from intronic regions of protein coding genes, sense which is transcribed in same direction and on same strand of neighboring coding genes; Both exonic and intronic sense lncRNAs are possible. Antisense which can be multiple exonic and intronic also but is transcribed from the reverse strand of neighboring coding gene. Bidirectional which is transcribed from region near to promoter of neighboring coding gene but from opposite strand. Enhancer which is transcribed from coding gene's enhancer region

LncRNAs are named after their biogenesis locations in relation to the coding genes [42], which is illustrated in Fig. 1. LncRNAs can be intergenic (lincRNAs) which are derived from gaps between genes, usually placed between protein-coding genes, intronic-lncRNAs which originate from a protein-coding genes' intronic regions, sense-lncRNAs which are produced from same strand and direction of neighboring protein-coding genes. On the other hand, the antisense-lncRNAs (aslncRNA) called natural antisense transcripts (NATs) are generated from transcription of complementary strands of protein-coding genes. Likewise, the bidirectional-lncRNAs which are derived from sequences that are close to protein-coding genes' transcription start sites, but from reverse strand also. Enhancer RNAs (eRNAs) which are generated from protein-coding genes' upstream enhancer and promoter regions [38, 43, 44].

### LncRNA structure

The biogenesis of lncRNAs is mediated by RNA polymerase II, similar to that of messenger RNA (mRNA) [45]. As a result, many lncRNAs have caps on the 5′ end and poly(A) tails on the 3′ end [46]. The majority of lncRNAs are thought to have more than two exons, as well as secondary and tertiary structures [47]. For each transcriptional start of a given lncRNA, nearly two distinct 3′ ends can be detected. Alternate cleavage and polyadenylation are the two processes that contribute to alternative 3′ ends, resulting in generation of different isoforms of lncRNAs from the same site, which can be increased even more by alternative splicing events [48, 49]. On the other hand, there is an exception in some lncRNAs which can be un-polyadenylated [38].

LncRNA-encoding genes generally have their own promoters, transcription factors (TFs) and distinctive DNA motifs, suggesting that transcription of lncRNAs may be an independent epigenetic modification [47]. Moreover, other epigenetic factors as DNA methylation can regulate lncRNAs transcription [47]. LncRNAs can be found in the nucleus, cytoplasm, as well as body vesicles such as exosomes and mitochondria [50]. More than half of the expressed lncRNAs are cytoplasmic, where they relate to polysome fractions, regulating mRNAs stability and translation [51].

### LncRNAs as epigenetic regulators

**Fig. 2:**
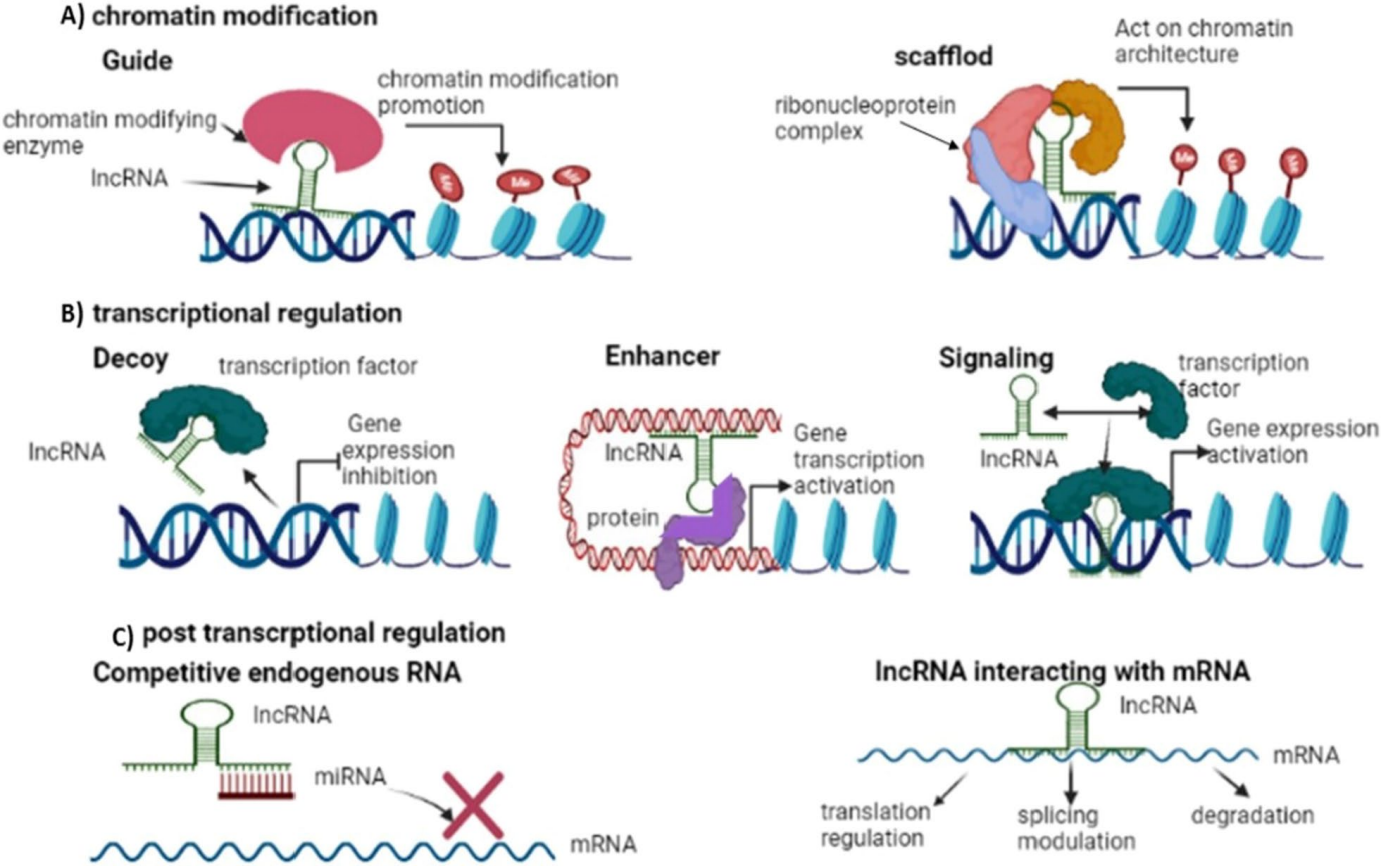
LncRNAs as epigenetic regulators. In **A** lncRNA can control gene expression via modfying chromatin architecture; guide lncRNA interacts with chromatin modfying enzyme firstly, then guiding it to specfic gene locus. While scaffold serves as platfrom for developing of RNP complexes through interaction with ribonucleoproteins. In **B** lncRNA can control gene transcription, Decoy lncRNA inhibits transcription by trapping TFs; while enhancer or signal lncRNAs activate transcription by acting as TF or TF activator respectively. In **C** lncRNAs can post transcriptionally control gene expression by acting as ceRNA which sponges other regulatory ncRNAs, miRNAs, preventing their interaction with target mRNA or via lncRNA direct interaction with mRNA

LncRNAs have the capacity to regulate several biological processes in both the normal and the disease states [52, 53]. LncRNAs play a key role in regulation of gene expression (54), which is clarified in Fig. 2.

LncRNAs can act as chromatin modifiers as guide lncRNA, interacting with chromatin-modifying-enzymes, mediating epigenetic modification by recruiting the developed chromatin remodeler complex to a specific gene locus [45]. On the other hand, scaffold lncRNA can assist in ribonucleoprotein (RNP) complexes assembly by interacting and placing proteins close to each other [29], Fig. 2A. And, depending on the proteins and RNAs present, transcriptional activation or repression is the result, once the complexes have been wholly developed [55]**.**

LncRNAs can act as transcriptional regulators, including decoy lncRNA which repress transcription of its neighboring coding gene by trapping regulatory factors including TFs [56]. Enhancer lncRNA which can function as a transcription factor-like molecule or enhancer, to boost gene expression [57]. Moreover, signal lncRNAs act as a molecular signal to control transcription in response to diverse stimuli [55]. As a result, its presence and synthesis can be used as a measure of transcriptional activity [55], Fig. 2B.

LncRNAs can act as post-transcriptional regulators, including competitive endogenous RNA (ceRNA) acting as sponge for miRNAs and hence, silencing its target mRNA [58] or lncRNAs-mRNA direct interaction via recognizing complimentary sequences, with an overall regulation of capping, splicing and mRNA stability [54], Fig. 2C.

### Notch-signaling mechanism (Fig. 3)

**Fig. 3:**
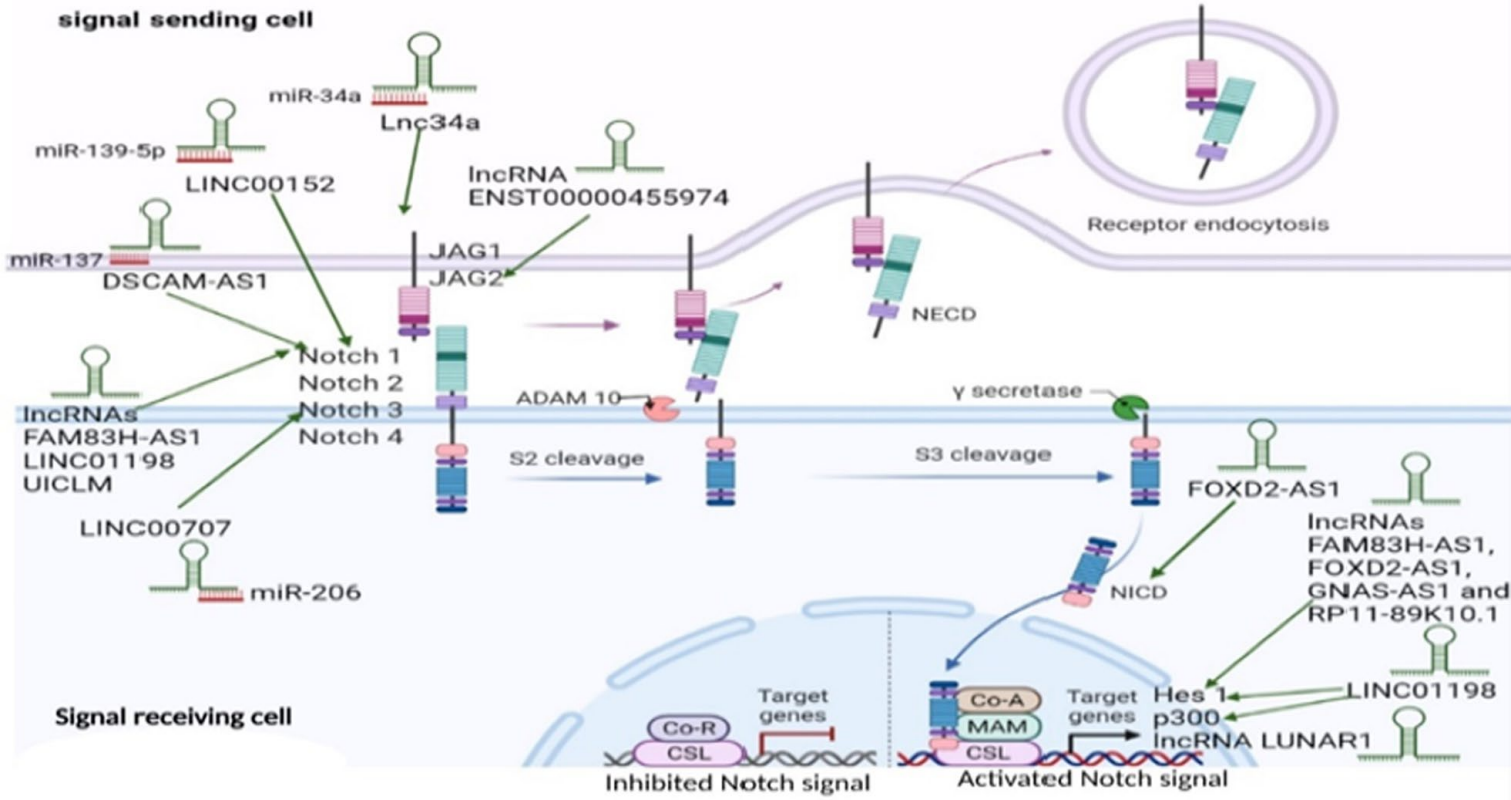
Notch-signaling and Notch-activation pathways in cancer, in relation to Notch associated-lncRNAs target genes. Notch receptors; Notch1, Notch2, Notch3, Notch4, Notch ligands; DLL; Delta-like ligand DLL1, DLL3, DLL4, and JAG, Jagged; JAG1, JAG2, ADAM; A Disintigrin and metalloproteases,, Co-R; co-repressors, co-A; co-activators, CSL transcription factor; (*CBF1* Suppressor of Hairless, Lag-1), *Hes1* hairy and enhancer of split-1, *MAM* mastermind proteins, *NICD* Notch intracellular domain, *NECD* Notch extracellular domain, *Ƴ secretase* gamma secretase. Notch signaling mechanism can be reinforced in cancer cells by lncRNAs, which can directly engage the Notch signal genes, boosting their expression, or disrupt miRNAs implicated in Notch signal suppression at specific stage

The binding of Notch ligand on one cell's membrane to a Notch receptor (Notch1, Notch2, Notch3) on the contacting cell's membrane initiates Notch signaling [59]. A two-step proteolysis cleavage process of Notch receptors starts once the ligand binds to them on the cell surface [60]. The ADAM enzymes (a disintegrin and metalloproteinase) catalyze the initial cleavage, resulting in the loss of the Notch's extracellular domain (NECD), which is then released by endocytosis [61]. While, the second cleavage is triggered by gamma-secretase, leading to release of the active Notch intracellular domain (NICD) [62, 63]. NICD enters the cell nucleus and interacts with the transcription factor CSL (CBF1, Suppressor of Hairless, Lag-1) and co-A activators mastermind (co-A MAM), all forming CSL-Notch-Mastermind transcription factor complex [62, 64], which is responsible for activating the Notch-target molecules transcription, like hairy and enhancer of split-1 (Hes1) and p300 [65]. Hes and Hey families' members are the most well-known Notch targets, which contribute to control many gene expression features related to cell fate regulation such as proliferation, differentiation and apoptosis [61]. The Hes family of transcription factors, specifically Hes1 in the gut, are the best identified Notch targets [66].

### Notch-associated-lncRNAs mechanism(s) profiling in cancer

Physiologically, several lncRNAs have been found to have a positive or negative association with the Notch-signaling pathway as well as micro-RNAs or mRNA as the Notch-related molecules [30]. In cancer generally, and colorectal specifically, activation of Notch-signaling can be influenced by several dysregulated lncRNAs, on the other hand, Notch-activation controls the expression of same or other lncRNAs, as depicted in Fig. 3. A list of lncRNAs interacting with the Notch-cascade contributing to various tumors development are presented in Tables 1 and 2.

**Table 1:** List of down and upregulated lncRNAs expressions in different cancers and their Notch-target gene(s)

Expression	LncRNA	Cancer type	Notch-target gene(s)	Refs.
Downregulated	LET	Non-small cell lung cancer (NSCLC)	Notch1 intracellular domain	[67]
	NBR2	NSCLC & Osteosarcoma	Notch1	[68, 69]
	MEG3	Endometrial	Notch 1, Hes1	[70]
	MIR22HG	Gastric	Notch2 signaling	[71]
	LincRNA-p21	Hepatocellular	Hes1 and NICD	[72]
	LINC00261	Hepatocellular	Notch1 and Hes1	[73]
	CEBPA-AS1	Osteosarcoma	Hes1 and RBPJ	[74]
	RAMP2-AS1	Glioblastoma	Notch3	[75]
	HCG18	Bladder	Notch1	[76]
	PAUPAR	Uveal melanoma	Hes1	[77]
Upregulated	Xist	NSCLC & Pancreatic	Notch1 via sponging miR-137	[78, 79]
	Lbx2-as1	NSCLC	Notch1, p21, Hes1	[80]
	PVT 1	NSCLC	Notch1, NICD and HES1 via YAP1 activation	[81]
	LINC01783	NSCLC	DLL-1 via targeting miR-432-5p	[82]
	NALT1	Gastric & Leukemia	Notch 1	[83, 84]
	SNHG1	Gastric	Notch1 via sponging miR-15b	[85]
	Linc01555	Gastric	Notch1, Notch2, DLL3 and Hes1	[86]
	DLEU2	Gastric Cervical	Notch2 via sponging miR-23b-3p Notch1 and RBPJ through impeding p53 expression	[87] [88]
	SRA	Cervical	Notch1, Hes1 and p300	[89]
	DARS-AS1	Cervical	JAG1 via sponging miR-628-5p	[90]
	HOTAIR	Retinoblastoma Cervical Pancreatic	Notch1 and JAG1 Notch1, Hes1and P300 Notch3 via sponging miR-613	[91] [92] [93]
	ROR	Retinoblastoma Endometrial	Notch1 via sponging miR-32 Notch1 via regulating miR34a expression	[94] [95]
	GHET1	Prostate	Notch1, HIF-1α via negative regulation of KLF2	[96]
	FEZF1-AS1	Prostate NSCLC & Glioblastoma	Notch1, p21 and Hes1 Notch1 via sponging miR-34a	[97] [27, 98]
	DANCR	Prostate	JAG1 via sponging miR-34a-5p	[99]
	Linc-OIP5	Glioma Breast	Jag1, Notch1 and Hes1 JAG1	[100] [101]
	ZFAS1	Glioma	Hes-1 and NICD	[102]
	LINC01152	Glioblastoma	Notch-pathway via MAML2 + ve regulation	[103]
	PlncRNA-1	Glioma	Notch1, JAG1 and Hes1	[104]
	LINC01410	Glioma	Notch2	[105]
	SNHG3	Breast	Notch; competitively binding miR-154-3p	[28]
	SNHG7	Breast	Notch 1 via sponging miR-34a	[106]
	SNHG12	Osteosarcoma Nasopharyngeal	Notch2 via sponging miR195-5p Notch pathway	[107] [108]
	CRNDE	Osteosarcoma	Notch1, JAG1 and EMT related proteins	[109]
	RP11–567G11.1	Pancreatic Renal	Jagged1, Hes1, Hes5 and Math1 Jagged1, Hes5 and Hey1	[110] [111]
	HCG11	Pancreatic	NICD and Hes1 via sponging miR-579-3p	[112]
	LNCRNA00673	Hepatocellular	Notch1 and Notch3	[113]
	UCA1	Tongue	JAG1 and Notch1 via sponging miR-124	[114]
	MALAT1	Ovarian	Notch1 pathway	[115]
	DLX6-AS1	Epithelial ovarian	Notch1, p21 and Hes1	[116]
	LncND	Neuroblastoma	Notch1 & Notch2 via sponging miR-143-3p	[117]
	HNF1A-AS1	Oral squamous cell	Notch1, Hes1	[118]
	CCAL	Papillary thyroid	Notch1 signaling	[119]
	LINC01123	Lung adenocarcinoma	Notch1 via sponging miR-449b-5p	[120]
	BANCR	Melanoma	Notch2 via sponging miR‑204	[121]
	Linc00152	Infantile hemangioma	Notch1, Hes1 and Hey1	[122]
	MEG8	Hemangioma	Notch2 via sponging miR-497-5p	[123]
	HOXA-AS2	Cervical	NICD	[124]

**Table 2:** List of cancer types with lncRNAs expressions down and upregulated and their Notch-target gene(s)

Cancer	LncRNA	Expression	Notch-target gene(s)	Refs.
NSCLC	LET NBR2	Downregulated	Notch1 intracellular domain Notch1	[67] [69]
	Xist Lbx2-as1 PVT1 LINC01783 FEZF1-AS1	Upregulated	Notch1 via sponging miR-137 Notch1, p21 and Hes1 Notch1, NICD and Hes1 via YAP1 activation DLL-1 via targeting miR-432-5p Notch1 via sponging miR-34a	[79] [80] [81] [82] [27]
Lung adenocarcinoma	LINC01123	Upregulated	Notch1 via sponging miR-449b-5p	[120]
Osteosarcoma	NBR2 CEBPA-AS1	Downregulated	Notch1 Hes1 and RBPJ	[68] [74]
	SNHG12 CRNDE	Upregulated	Notch2 via sponging miR195-5p Notch1, JAG1 and EMT related proteins	[107] [109]
Endometrial	MEG3	Downregulated	Notch1, Hes1	[70]
	ROR	Upregulated	Notch1 via regulating expression of miR34a	[95]
Gastric	MIR22HG	Downregulated	Notch2 signaling	[71]
	NALT1 SNHG1 Linc01555 DLEU2	Upregulated	Notch1 Notch1 via sponging miR-15b Notch1, Notch2, DLL3 and Hes1 Notch2	[83] [85] [86] [87]
Hepatocellular	LincRNA-p21 LINC00261	Downregulated	NICD and Hes1 Notch1 and Hes1	[72] [73]
	LNCRNA00673	Upregulated	Notch1 and Notch3	[113]
Glioma	RAMP2-AS1	Downregulated	Notch3	[75]
	FEZF1-AS1 Linc-OIP5 ZFAS1 LINC01152 PlncRNA-1 LINC01410	Upregulated	Notch1via sponging miR-34a JAG1, Notch1 and Hes1 Hes1 and NICD Notch pathway Notch1, JAG1 and Hes1 Notch2	[98] [100] [102] [103] [104] [105]
Bladder	HCG18	Downregulated	Notch1	[76]
Pancreatic	Xist HOTAIR RP11-567G11.1 HCG11	Upregulated	Notch1 via sponging miR-137 Notch3 via sponging miR-613 JAG1, hes1, hes5 and MATH1 NICD and Hes1 via sponging miR-579-3p	[78] [93] [110] [112]
Melanoma	PAUPAR	Downregulated	Hes1	[77]
	BANCR	Upregulated	Notch2 via sponging miR-204	[121]
Cervical	DLEU2 SRA DARS-AS1 HOTAIR HOXA-AS2	Upregulated	Notch1 and RBPJ through impeding p53 expression Notch1, Hes1 and p300 JAG1 via sponging miR-628-5p Notch1, Hes1 and p300 NICD	[88] [89] [90] [92] [124]
CRC	FOXD2-AS1 FAM83H‑AS1 LINC00152 DSCAM-AS1 LINC01198 LINC00707 ENST00000455974 GNAS-AS1 & RP11-89K10.1 Lnc34a UICLM	Upregulated	NICD, Hes1 Notch1 and Hes1 Notch1 via sponging miR-139-5p Notch1 via sponging miR-137 Notch1, p300 and Hes1 Notch3 & TM4SF1via sponging miR-206 JAG2 Hes1 Notch pathway via sponging miR-34a Notch1	[125] [126] [127] [128] [129] [130] [131] [132] [133, 134] [135]
Breast	Linc-OIP5 SNHG3 SNHG7	Upregulated	JAG1 Notch by binding to miR-154-3p Notch1via sponging miR-34a	[101] [28] [106]
Retinoblastoma	HOTAIR ROR	Upregulated	Notch1, JAG1 Notch1 via sponging miR-32	[91] [94]
Prostate	GHET1 FEZF1-AS1 DANCR	Upregulated	Notch1 and HIF-1 α via negative regulation of KLF2 Notch1, p21 and Hes1 JAG1 via sponging miR-34a-5p	[96] [97] [99]
Tongue	UCA1	Upregulated	JAG1 and Notch1 via sponging miR-124	[114]
Ovarian	MALAT1 DLX6-AS1	Upregulated	Notch1 pathway Notch1, p21 and Hes1	[115] [116]
Renal	RP11-567G11.1	Upregulated	JAG1, hes5 and Hey1	[111]
Acute leukemia	NALT1	Upregulated	Notch1	[84]
Nasopharyngeal	SNHG12	Upregulated	Notch pathway	[108]
Neuroblastoma	LncND	Upregulated	Notch1&Notch2 via sponging miR-143-3P	[117]
Oral squamous	HNF1A-AS1	Upregulated	Notch1 and hes1	[118]
Hemangioma (infantile)	MEG8 Linc00152	Upregulated	Notch2 via sponging miR-497-5p Notch1, Hes1 and hey1	[123] [122]
Papillary thyroid	CCAL	Upregulated	Notch1 signal	[119]

LncRNA-low expression in tumor (lncRNA-LET) a newly discovered lncRNA, was detected on chromosome 15q24.1 [136]. In NSCLC, LET demonstrated a tumor-suppressive effect; its overexpression in cells decreased NICD1 level [67]. As well, Neighbor of *BRCA1 gene 2* (NBR2) is lncRNA that is encoded from the gene which locates near to the tumor suppressor gene *BRCA1* [137]. NBR2 acts as tumor suppressor by inhibiting Notch1 expression in NSCLC and osteosarcoma [68, 69]. Additionally*, Maternally expressed gene 3 (MEG3)* is an imprinted gene in humans locating on chromosome 14q32.3, encodes lncRNA MEG3 [138]. lncRNA MEG3 inhibits endometrial tumor growth by negatively regulating Notch1 and Hes1 levels [70].

*Human miR-22 host gene (MIR22HG)* is a tumor suppressor lncRNA stimulating the expression of miR-22 [139]. *MIR22HG* suppresses Notch2 signaling, inhibiting progression of gastric cancer [71]. Likewise, LincRNA-p21 is 15 kb upstream from *p21 gene*, that can control both mRNA translation as well as protein stability [140]. LincRNA-p21 enhanced level reduces expression of Notch proteins, Hes1 and NICD, inhibiting hepatocellular carcinoma invasion and metastasis [72]. Moreover, CCAAT Enhancer Binding Protein Alpha (CEBPA) is a transcription factor that can regulate cell cycle with oncogenic functions [141]. CEBPA-AS1 is the CEBPA antisense RNA1 [142]. CEBPA-AS1 attenuates osteosarcoma progression via inhibiting Notch pathway members, Hes1 and RBPJ [74].

Receptor activity modifying protein 2 (RAMP2) is a single-transmembrane domain protein that plays key role in endothelial homeostasis. lncRNA RAMP2-AS1 is transcribed from RAMP2 antisense [143]. RAMP2-AS1 overexpression in cells represses Notch3 expression, impeding glioblastoma progression [75]. Besides, *PAX6* upstream antisense RNA (PAUPAR) is lncRNA that could control expression of its adjacent gene *Pax6*, a transcription factor which controls neuronal differentiation [144]. PAUPAR serves as tumor suppressor in uveal melanoma via negatively regulation of Hes1 expression [77].

Xist (X inactive specific transcript) is the key regulator of X chromosome inactivation, which results in the stable and reliable one X chromosome silencing in somatic cells of female mammals in early development stages [145]. Xist acts as oncogenic lncRNA in NSCLC & pancreatic cancer via sponging miR-137, promoting Notch1 expression [78, 79]. Additionally, *Ladybird-like homeobox gene 2 (LBX2)* is a transcription factor encoding gene located on chromosome 2p13.1, involved in regulation of heart development as well as tumorigenesis of CRC [146]. LBX2-AS1, *LBX2* antisense1 is lncRNA transcribed from an intron of the same chromosome [147]. In NSCLC, LBX2-AS1 functions as tumor promoter that positively regulates Notch signal markers, Notch1, p21 and Hes1, expressions [80].

Plasmacytoma variant translocation 1 (PVT1) lncRNA was firstly discovered in murine leukemia virus-induced T lymphomas as a ubiquitous retroviral integration site; PVT1 acts as oncogenic lncRNA in many cancers [148]. Upregulation of PVT1 promotes NSCLC progression through Yes-associated protein 1 (YAP1) mediated Notch pathway activation, boosting Notch1, NICD and Hes1 levels [81]. Likewise, Notch 1 associated lncRNA in T cell acute lymphoblastic leukemia1 (NALT1*)* is identified to cis-regulate its neighboring gene, Notch1, supposing that NALT1 actions is relayed on Notch signaling [83]*.* NALT1 overexpression activates Notch 1 expression in both gastric cancer and pediatric T cell acute lymphoblastic leukemia [83, 84].

*Small nucleolar host gene 1* (SNHG1) is host for 8 small nucleolar RNAs, which contributes to ribosomal RNA modifications [149]. Overexpressed SNHG1 positively regulates Notch 1 and Doublecortin-like kinase 1 (DCLK1) expressions via modulation of miR-15b, inducing EMT in gastric cancer [85]. Besides, *Deleted in Lymphocytic Leukemia 2 (DLEU2)* is an RNA gene which is firstly discovered in chromosome 13q14 genomic region, a region that is usually eliminated in B-cell chronic lymphocytic leukemia [150]. *DLEU2* promotes gastric cancer progression via serving as ceRNA for miR-23b-3p enhancing Notch2 expression [87]. While, upregulated *DLEU2* induces cervical cancer proliferation by inhibition Notch pathway activity, Notch1 and RBPJ, through impeding p53 expression [88].

Steroid receptor RNA activator (SRA) is lncRNA that can activate transcriptional activity of steroid receptor [151]. SRA upregulation contributes to cervical tumorigenesis through enhancing Notch signal, promoting Notch1, Hes1 and p300 levels [89]. Additionally, lncRNA DARS antisense RNA 1 (DARS-AS1) that can also regulate its neighboring gene *DARS* (aspartyl-tRNA synthetase) is identified as tumor enhancer in various cancers [152]. DARS-AS1 enhanced expression positively regulates JAG1 through sponging miR-628-5p, inducing cervical tumorigenesis via Notch activation [90].

HOX transcript antisense RNA (HOTAIR) is lncRNA, transcribed from antisense strand of *Homeobox C (HOXC)* cluster gene; *HOX* genes encode essential embryonic development regulators. HOTAIR is a crucial regulator of chromatin structure and organization that controls expression of *HOXD* cluster genes [153, 154]. HOTAIR serves as tumor promoter, increasing Notch1 and JAG1 expressions in retinoblastoma [91]. Also, HOTAIR overexpression increases Notch markers levels, Notch1, Hes1 and P300, enhancing cervical carcinogenesis [92]. Moreover, HOTAIR positively regulates Notch 3 through serving as ceRNA for miR-613 in pancreatic cancer [93].

Regulator of reprogramming (ROR) is promoter lncRNA for reprogramming of pluripotent stem cells. ROR is a key player in human embryonic stem cells self-renewal and differentiation [155]. ROR higher levels activates Notch1 expression via negative regulation of miR-32, stimulating EMT in retinoblastoma [94]. While ROR enhances Notch1 expression in endometrial cancer via regulating of miR34a [95]. As well, gastric carcinoma highly expressed transcript 1 (GHET1) is a confirmed oncogene lncRNA in multiple tumors [156]. Upregulated GHET1 increases prostate cancer proliferation through inducing HIF-1α and Notch 1 signal via negative regulation of Kruppel-like factor 2 (KLF2) [96].

FEZF1-AS1 is FEZ family Zinc Finger 1-Antisense RNA 1, a novel oncogenic lncRNA in various tumors [157]. FEZF1-AS1 is a tumor promoter in prostate cancer via Notch signal activation, overexpressed FEZF1-AS1 contributes to higher levels of Notch1, p21 and Hes1 [97]. On the other hand, FEZF1-AS1 upregulates Notch1 in NSCLC and glioblastoma via negative regulation of miR-34a [27, 98]. Besides, Differentiation antagonizing non-protein coding RNA (DANCR) is a lncRNA prevents differentiation of epidermal progenitor cells into osteoblasts [158]. DANCR overexpression positively regulates JAG1 via targeting miR-34a-5p, aggravating prostatic cancer resistance to docetaxel [99].

Linc-OIP5 (opa interacting protein5) is identified to regulate neurogenesis [159]. Linc-OIP5 overexpression enhances glioma tumorigenesis through Notch activation, upregulating JAG1, Notch-1 and Hes1 expressions [100]. Also, Linc-OIP5 knockdown in breast cancer cells was highly associated with JAG1 expression downregulation [101]. On the other hand, Zinc Finger NFX1-Type Containing 1 (ZNFX1) antisense RNA (ZFAS1) is an lncRNA transcribed from antisense strand next to ZNFX1 protein coding gene; ZFAS1 is identified as a regulator of alveolar and epithelial cell development in the mammary gland [160]. ZFAS1 serves as tumor promoter in glioma cells through upregulating of Notch pathway, enhancing Hes-1 and NICD levels [102]. Likewise, Prostate cancer-up-regulated RNA 1 (PlncRNA-1) is lncRNA transcript that is firstly recognized to be overexpressed in prostate cancer [161]. PlncRNA-1 overexpression induces glioma progression through boosting expressions of Notch1, JAG1 and Hes1, stimulating Notch signal [104].

Colorectal Neoplasia Differentially Expressed (CRNDE) lncRNA exhibits tissue-specific and time-specific expression patterns, is firstly discovered with its upregulated expression in colorectal adenomas and carcinomas [162]. overexpressed CRNDE lncRNA functions as oncogene in osteosarcoma cells via upregulation of Notch1, JAG1 and EMT related proteins [109]. Additionally, Urothelial carcinoma associated 1 (UCA1) is a lncRNA that is primarily discovered from cell lines of bladder cancer [163]. UCA1 elevated level positively regulates JAG1 and Notch1 through targeting miR-124, promoting tongue carcinoma [114].

Metastasis associated in lung adenocarcinoma transcript 1 (MALAT1) lnRNA is originally recognized in non-small-cell lung cancer (NSCLC) primary stages study, so was given that name. MALAT1 clinical relevance is related to metastasis prediction and survival in NSCLC early stages [164]. By blocking the Notch1 signalling pathway, MALAT-1 knockdown increased the chemosensitivity of ovarian cancer cells to cisplatin [115]. Besides, Distal-less homeobox 6 antisense 1 (DLX6-AS1) is a lncRNA expressed in normal brain tissue [165]. Upregulated DLX6-AS1 is epithelial ovarian tumor enhancer through modulating of Notch signal, DLX6-AS1 silencing was highly associated with decreasing levels of Notch1, p21, and Hes1 [116].

LncRNA for neurodevelopment (LncND) is linked to a neuro-developmental diseases in humans [117]. LncND overexpression upregulates Notch1 & Notch2 in neuroblastoma cells through inhibiting of miR-143-3p [117]. On the other hand, Hepatocyte nuclear factor-1 homeobox A (HNF1A) antisense RNA 1 (HNF1A-AS1) lncRNA is transcribed in the reverse direction of the *HNF1A* gene [166]. HNF1A-AS1 increased expression stimulates Notch pathway in oral squamous cell carcinoma; suppression of HNF1A-AS1 inhibits the expression of Notch1 and Hes1 [118].

Colorectal cancer-associated lncRNA (CCAL) has a verified oncogenic functions in CRC cells [167]. Upregulated CCAL activates Notch1 pathway, which aided in the progression of papillary thyroid cancer [119]. Additionally, *BRAF*-activated non-coding RNA (BANCR) is lncRNA discovered in melanoma cells at first, then its aberrant expression was verified in several cancers including CRC [168]. In melanoma, BANCR is a tumor promoter, negatively targets miR‑204, enhancing Notch 2 expression [121].

HOXA cluster antisense RNA2 (HOXA-AS2) lncRNA is transcribed from region between *HOXA3* and *HOXA4* regions [169]. In cervical cancer cells, HOXA-AS2 triggers Notch signal, downregulation of HOXA-AS2 contributes to decreased NICD protein level as well as significantly reducing interaction between NICD and the transcription factor RBP-JK [124].

### Molecular mechanisms driving CRC in relation to Notch-Signaling

**Fig. 4:**
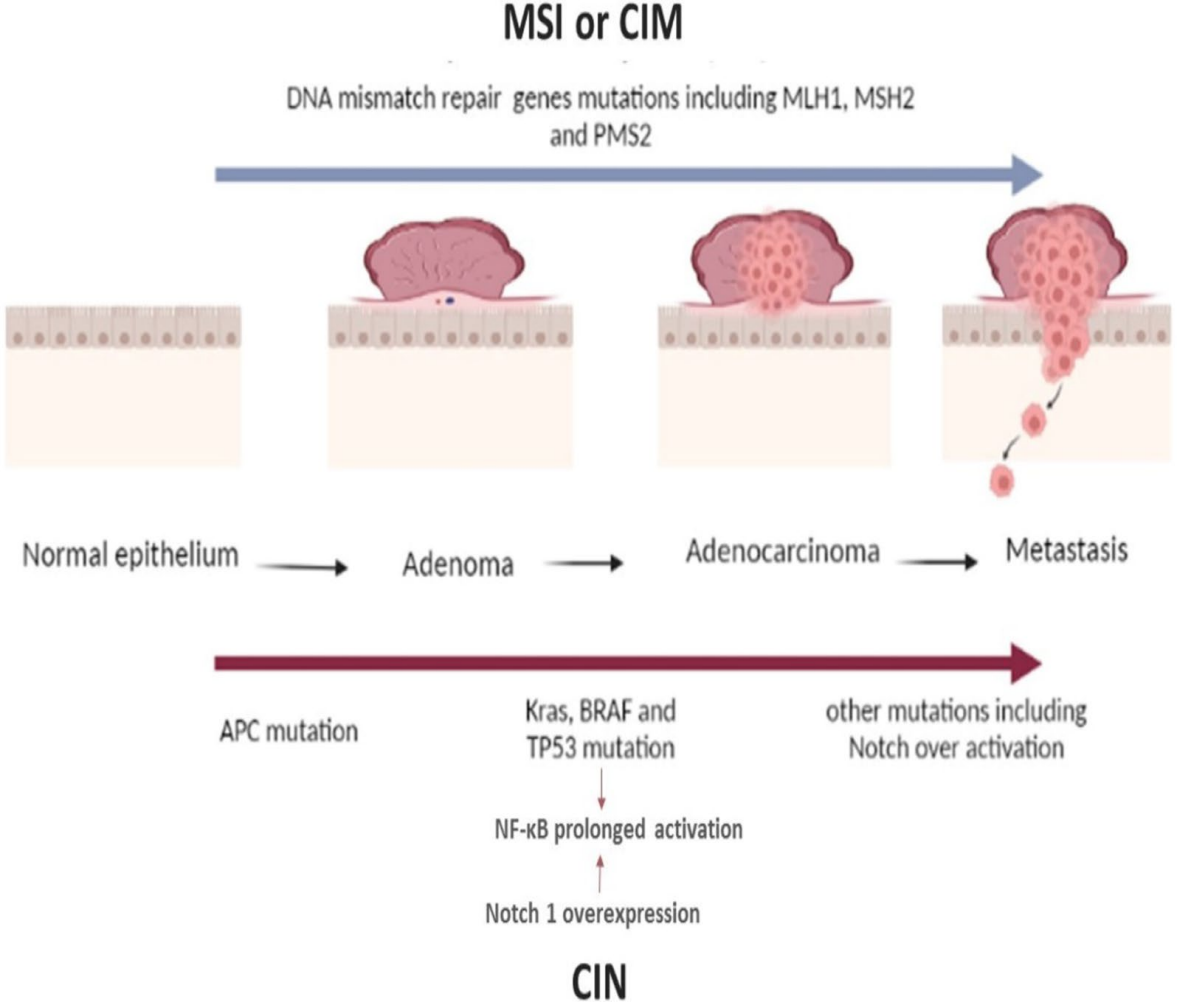
Molecular mechanisms driving CRC in relation to Notch-signaling. *APC* adenomatous polyposis coli, *BRAF* v-raf murine sarcoma viral oncogene homolog B1, *CIM* CpG island methylation, *CIN* Chromosomal instability, *Kras* Ki-ras2 Kirsten rat sarcoma viral oncogene homolog, *MSI* microsatellite instability, *NF-κB* nuclear factor kappa B cell, *TP53* tumor protein p53

Mechanisms accompanied by oncogene(s) activation and inhibition of tumor suppressors expression [5], though driving CRC formation as in Fig. 4 are chromosomal instability (CIN), CpG island methylation (CIM), and microsatellite instability (MSI) [170].

The conventional chromosomal instability mechanism is characterized by the accumulation of mutations that are initiated after mutational inactivation in the adenomatous polyposis coli (APC), accompanied by oncogenes activations including ki-ras2 Kirsten rat sarcoma viral oncogene homolog (Kras), cyclooxygenase-2 (COX2) and v-raf murine sarcoma viral oncogene homolog B1 (BRAF), tumor suppressor genes silencing including TP53, Deleted in colon cancer/Deleted in pancreatic cancer locus4 (DCC/DPC4) and loss of heterozygosity of chromosome 18 [171, 172].

Microsatellite instability is caused by faulty or inactive DNA mismatch repair genes (MMR). Mutations in MMR genes create genetic abnormalities in other tumor suppressor genes that are associated with growth control [171]. Hereditary non-polyposis CRC syndrome and Lynch syndrome are characterized by microsatellite instability, resulting from one of MMR genes hereditary mutations such as *MLH1, MSH2,* and *PMS2* [170, 173].

CpG island methylation pathway is identified by hypermethylation of CpG islands in gene promotors, contributing to transcriptional inhibition of varies tumor suppressor genes including MMR genes [173].

It is noteworthy to mention that inflammation-driven CRC promoted by a mutant version of the tumor protein 53 (p53(, is mediated via the nuclear factor kappa B cell (NF-κB) prolonged activation [174]. NF-κB activation is enhanced by Notch1 overexpression, which upregulates the transcriptional activity of NF-kappa-B p65 subunit [175]. Furthermore, inactivation of TP53 enhances the progression of Notch-induced invasive adenocarcinoma (in the glandular tissue) with EMT characteristics [176]. In other words, we can infer that combination of Notch1 hyperactivity with oncogenic Kras activation and TP53 inactivation promotes high rates of metastasis of intestinal adenocarcinoma [17] (Fig. 4).

### Notch signaling as a regulator of CRC immune response

Substantial evidence supports the critical function of the Notch pathway in the immune system [177]. Notch signal controls the activation of, CD8^+^ cytotoxic T cell, which is the key player in the anti-tumor immunological function [178]. Additionally, the crosstalk between tumor cells and dendritic cells, which is necessary for the generation and proliferation of T regulatory (Treg) cells in the TME, is significantly influenced by JAG1-induced Notch activation [179]. JAG1-Notch3 signal activation has been revealed to be crucial for Treg generation and expansion driven by OX40 ligand [180]. Besides, JAG1-mediated maturation of dendritic cells encourages Treg survival and proliferation [181].

In CRC, both peripheral blood samples and tissues showed increased Notch1, Hes1 and Hes5 mRNA expressions in CD8^+^ T cells, while Notch2 mainly displayed enhanced level in tissue specimens [182]. Notch signal has potential immunosuppressive effect, which inhibits CD8^+^ T lymphocytes' cytolytic and noncytolytic activities by inducing programmed cell death protein-1 (PD-1). Silencing the Notch pathway enhances the cytotoxicity of tumor-infiltrating CD8^+^ T cells via increasing their production of pro-inflammatory cytokines such as interferon gamma (IFN-γ), interleukin-1β (IL-1β), IL-6, IL-8, tumor necrosis factor alpha (TNF-α), and vascular endothelial growth factor (VEGF) as well as reducing their PD-1 expression [182]. Likewise, it was showed that mutations in the Notch system were related with a rise in amount of tumor CD8^+^ T cells and a decline in Treg cells, with increasing expressions of immune checkpoints, chemokines and some effector molecules [183]. Notch mutation- induced immune checkpoints upregulation can stimulate better anti-tumor immunological response, suggesting that patients with these mutations may be more responsive to immune checkpoint blockades, which is a promising therapeutic approach intended to restore anticancer immune responses [183, 184].

Moreover, over activation of Notch1 signal drives metastasis in CRC in neutrophil-dependent manner via promoting chemokine CXCL5 (C-X-C Motif Chemokine Ligand 5) and transforming Growth Factor β (TGF-β) productions, triggering an inhibitory strategy to suppress T cell responses in the TME and create an immune-suppressed environment [17]. Additionally, accumulation of myeloid derived CD11b expressing cells in regions, where cells experienced EMT, requires Jag2 expression stimulating Notch signal and EMT [185]. Furthermore, it has been shown that measuring the proportion of circulating CD11b-Jag2 cells in patients may provide a sign of CRC development into a metastatic state [185].

### Mechanism(s) by which upregulated-Notch-associated lncRNAs cause CRC (Table 3)

**Table 3:** Mechanisms by which upregulated-Notch-associated lncRNAs cause CRC

Notch-associated lncRNA	Mechanism(s) driving CRC	Refs.
LINC00152	promotes cell proliferation, growth, invasion & migration & progression	[127]
LINC00707		[130]
DSCAM-AS1		[128]
FOXD2-AS1		[125]
ENST00000455974		[131]
GNAS-AS1 & RP11-89K10.1		[132]
UICLM	promotes CRC proliferation, growth, invasion, migration & regulates stemness	[135]
Lnc34a	promotes cancer stem cells self-renewal & CRC progression	[133, 134]
FAM83H‑AS1	promotes cell cycle progression, cell proliferation, invasion, migration & inhibits apoptosis	[126]
LINC01198		[129]
LUNAR1		[188]

LINC00152 named cytoskeleton regulator (CYTOR) was an overexpressed lncRNA in CRC tissues, acting via sponging miR-139-5p, leading to positive regulation of Notch1 expression. knockdown of Notch1 expression was shown to effectively inhibit CRC cell growth caused by LINC00152 upregulation, proving evidence that activities of LINC00152 are relayed on Notch1 activation [127].

Likewise, LINC00707 expression was highly elevated in CRC tissues, to sponge miR-206 and regulate expression of its target molecules, Notch3 and transmembrane 4 L six family member 1 (TM4SF1) [130].

Additionally, lncRNA Down syndrome cell adhesion molecule Antisense RNA 1 (DSCAM-AS1) is a tumor promoter that is upregulated in CRC tissues. DSCAM-AS1 positively regulates Notch1 through targeting miR-137. Where, the inhibitory effects caused by silencing of DSCAM-AS1 in CRC cells, could be reversed by Notch1 overexpression or miR-137 suppression [128].

Besides, LncRNA FOXD2 adjacent opposite strand RNA 1 (FOXD2-AS1) was significantly overexpressed in CRC tissues. Inhibition of FOXD2-AS1 expression in CRC cells resulted in an enhancement of E-cadherin protein expression, decreasing the expression of N-cadherin and the Snail protein as well as significant decrement in the Notch-related proteins (NICD and Hes-1) expression, suggesting that FOXD2-AS1 promoted CRC progression through regulation of EMT and Notch pathway [125].

In addition, ENST00000455974 is an upregulated lncRNA in DNA mismatch repair-proficient colon cancer tissues. ENST00000455974 can regulate both mRNA and the Notch-signaling ligand JAG2 protein expression [131].

Moreover, Yuqin Zhang et al., verified that lncRNAs GNAS Antisense RNA 1 (GNAS-AS1) and RP11-465L10.10 expressions were significantly elevated in CRC tissues, being involved in CRC development through direct binding to the Notch downstream target Hes1^132^. Hes1 is involved in CRC stem cells self-renewal and tumorgenicity, promoting cell proliferation and migration [186, 187].

Furthermore, the expression of up-regulated in CRC liver metastasis (UICLM) lncRNA was elevated in CRC tissues, when being silenced, contributing to the downregulation of essential stemness-related-genes, including Notch1 [135].

In addition, the lncRNA-34a (Lnc34a) is increased in CRC and epigenetically suppresses miR-34a expression [133].

Besides, the expression of lncRNA FAM83H antisense RNA1 (FAM83H‑AS1) was enhanced in CRC tissues, and when was knocked down in CRC cell lines, resulted in suppression in both mRNA and Notch1 and Hes1 protein levels, countered through the Notch-signal activator JAG1 [126].

LINC01198 was an another upregulated lncRNA in CRC tissues that regulates Notch-pathway markers, namely, Notch1, p300 and Hes1 [129].

Moreover, Leukemia-Associated Non-Coding IGF1R Activator RNA 1 (LUNAR1), a novel Notch-regulated lncRNA, was recently reported to be significantly upregulated in CRC tissues [188, 189], induced by Notch1 activation, enhancing CRC progression through sustaining insulin-like growth factor 1 receptor (IGF-1R) expression [188].

### Notch-associated lncRNAs impact on CRC clinical outcome (Table 4).

**Table 4:** Notch-associated lncRNAs impact on CRC clinical outcome

Notch-associated lncRNA	CRC clinical outcome				Refs.
	Size, TNM; Tumor stage	Disease-free survival/ OS	Recurrence/ Metastasis	Hazard ratio (HR)	
FOXD2-AS1	–	Poor OS	–	2.245	[190]
FAM83H‑AS1	Larger size ≥ 5 cm, advanced stage III-IV	Poor OS	–	1.542; 95% confidence interval (CI) (1.115–2.135)	[126, 192]
LINC00152	advanced stage III-IV	Poor disease-free survival & OS	Recurrence in Oxaliplatin-receiving patients	3.825; 95%(CI) (1.723–8.493)	[127, 194]
LINC00707	Larger size ≥ 5 cm, advanced stage III-IV	Poor OS	Lymphatic metastasis & distant metastasis	4.255; 95%(CI) (1.560–11.610)	[130, 196]
DSCAM-AS1	Advanced stage III-IV	Poor OS	Metastasis	–	[128]
ENST00000455974	–	Poor progression free survival		14.404; 95% (CI) (1.785, 116.242)	[131]
UICLM	Larger size, advanced stage III-IV	Worse progression free survival	Liver metastasis	2.13; 95%(CI) (1.77–3.06)	[135]
LUNAR1	Advanced stage III-IV	Unfavorable disease-free survival & OS	TNM	3.25; 95%(CI) (1.98–5.31)	[188]

Upregulation of FOXD2-AS1 is a predictor for poor survival in CRC, where FOXD2-AS1 higher expression was associated with clinical lower survival rate [190]. Furthermore, 12-year follow-up study after surgery, the survival rate analysis showed patients with enhanced FOXD2-AS1 expression, significantly exhibited 6-year survival rate, not 12-year, relative to those with lower expression [191].

FAM83H‑AS1 is an independent prognostic indicator in colon cancer. Where, patients with greater level of FAM83H‑AS1 had shorter OS time compared to patients with lower level [192]. Furthermore, FAM83H‑AS1 higher levels were significantly associated with larger tumor size and advanced tumor stage [126].

Additionally, overexpression of LINC00152 was associated with poor CRC prognosis, advanced tumor stage and worse OS as well as disease-free survival [127, 193]. Moreover, Linc00152 can be used as predictor for response of Oxaliplatin-receiving-patients, after radical colectomy, where high Linc00152 expression in Oxaliplatin-receiving-patients was associated with an increased N stage, recurrence, shorter OS and recurrence-free survival in comparison to patients with lower expression [194].

Likewise, LINC00707 and DSCAM-AS1 elevated levels are associated with poor patients' prognosis, shorter OS relative to those with lower expression [128, 195, 196]. LINC00707 enhanced expression was positively correlated with larger tumor size, advanced TNM stage, lymphatic metastasis and distant metastasis [130, 196].

DSCAM-AS1 upregulation was correlated to advanced clinical stage and metastasis status [128].

Besides, patients with higher levels of ENST00000455974 or UICLM had worse progression-free survival [131, 135]. Higher levels of UICLM were significantly correlated with CRC larger tumor size, advanced tumor stage as well as liver metastasis [135].

Again, LUNAR1 upregulation is associated with aggressive CRC, advanced tumor stage, poor differentiation status (high grade and stage), deeper tumor invasion and TNM, being attributed to unfavorable disease-free survival as well as OS [188].

### Notch-associated lncRNAs in relation to multi-drug resistance (MDR) in CRC (Table 5)

**Table 5:** Notch-associated lncRNAs in relation to multi-drug resistance in CRC

Notch-associated lncRNA	Multi-drug resistance in CRC				Refs.
	Chemotherapy used	miR sponged	Target protein(s)	Effect on apoptosis	
LINC00152	Oxaliplatin	miR-193a-3p miR-139-5p	-ERBB4 with AKT signal activation -Notch1 which induces upregulating of MRP-1 and BcL-2	Suppressed	[25, 127, 194]
LINC00707	5-FU topotecan, cisplatin & Astragaloside IV	miR-206	- Bcl-2 -Notch3	Lower apoptosis rate	[130, 199–201]
DSCAM-AS1	Oxaliplatin	miR-137	-YBX1 - Notch1 which induces upregulating MRP-1 and BcL-2	Inhibition of cytotoxicity	[25, 128, 203]
Lnc34a	5-FU	miR-34a	Lactate dehydrogenase	Inhibited	[133, 204]
ENST00000455974	Doxorubicin, 5-FU, Oxaliplatin	–	JAG2	Decreased apoptosis	[131, 205]
FAM83H‑AS1	5-FU	–	-Notch1 which induces upregulating MRP-1 & BcL-2 -Hes1 which induces upregulating ABCC1, ABCC2, P-gp1 & N-cadherin & suppressing of E-cadherin	Inhibited	[23, 25, 126]
UICLM	5-FU	–	Notch1 which induces upregulating MRP-1 & BcL-2	Inhibited	[25, 135]
FOXD2-AS1	5-FU	–	Hes1 which induces upregulating ABCC1, ABCC2, P-gp1 & N-cadherin & suppressing of E-cadherin		[23, 125]
GNAS-AS1 RP11-89K10.1	5-FU	–	Hes1 which induces upregulating ABCC1, ABCC2, P-gp1 & N-cadherin & suppressing of E-cadherin		[23, 132]
LINC01198	5-FU		-Notch1 which induces upregulating MRP-1 and BcL-2 -Hes1 which induces upregulating ABCC1, ABCC2, P-gp1 & N-cadherin &suppressing of E-cadherin	Inhibited	[23, 25, 129]
LUNAR1	5-FU		IGF-1R	Inhibited	[188, 210]

Cancer cells' adaptation reaction, to a diversity of cytotoxic drugs, is MDR, an obstacle to achieve effective chemotherapy [197].

Overexpression of linc00152 promoted colon cancer resistance to oxaliplatin-induced apoptosis. Linc00152 mediates drug resistance, through modulating erb-b2 receptor tyrosine kinase 4 (ERBB4) expression, by functioning as ceRNA negatively regulating miR-193a-3p expression with AKT (Protein kinase B) signaling activation [194]. Additionally, linc00152 overexpression inhibits 5-fluorouracil (5-FU) induced cell death in CRC, through activation of Notch1 and sponging miR-139-5p [127].

LINC00707 acts as ceRNA targeting miR-206 and inhibiting its expression, promoting its target Notch3 expression [130, 198]. MiR-206 downregulation, enhances resistance towards 5-FU by positively regulating B-cell lymphoma-2 (Bcl-2) protein level in CRC [199]. Additionally, downregulation of Notch3 in CRC cells was shown to improve the cells' chemosensitivity to topotecan as well as cisplatin with astragaloside IV coadministration [200, 201]. Therefore, LINC00707 may contribute to MDR through regulation of miR-206/Notch3 axis.

Likewise, DSCAM-AS1 targets miR-137 and negatively regulates its expression in CRC and breast cancer [128, 202]. Hence, downregulation of miR-137, promotes oxaliplatin-resistance via targeting Y-Box Binding Protein 1 (YBX1) [203].

Also, Lnc34a acts as ceRNA negatively regulating miR-34a expression [133]. miR-34a downregulation was shown to be associated with 5-FU resistance in colon cancer cells, via upregulation of its target protein levels lactate dehydrogenase [204].

Besides, ENST00000455974 positively regulates JAG2 expression. In CRC, JAG2 increased expression was shown to enhance chemoresistance to doxorubicin-induced cytotoxicity. Silencing JAG2, induced CRC cells apoptosis via suppression of p21 expression [205]. Additionally, knockdown of JAG2 expression was found to increase CRC cells chemosensitivity to 5-FU and oxaliplatin [205].

Moreover, studies identified that Notch1 could regulate MDR related genes multidrug resistance protein 1 (MRP1)/ ATP binding cassette subfamily C member 1 (ABCC1) and BcL-2 in cancer cells [206, 207]. In CRC, Notch1 overexpression is contributed to 5-FU resistance, Notch1 suppression via miR-139-5p overexpression increases 5-FU sensitization in CRC cells, depending on Notch1 downstream targets MRP-1 and BcL-2 downregulation [25]. Likewise, downregulation of Notch1 by miR-139-5p overexpression was associated with increasing drug sensitivity in MDR related to non-kinase transmembrane glycoprotein (CD44^+^) and CD133^+^ (prominin-1) CRC cells [208].

Furthermore, Hes1 elevated expression induces 5-FU resistance in CRC via enhancing EMT and ATP-binding cassette (ABC) transporters. Hes1 increased levels in CRC cells were highly associated with N-cadherin increasing expression, and E-cadherin suppressing expression which promote EMT [23]. Additionally, overexpression of Hes1 was contributed to upregulation of several ABC transporters, ABCC1, ABCC2 and p-glycoprotein 1 (P-gp1) which are crucial components in the metabolism of drugs [23]. ABC transporters are regarded as primary cause of treatment failure via reducing drug uptake and accumulation in cells. Resistance to a wide spectrum of anticancer medicines is conferred by overexpression of the ABCC1 transporter [23, 209].

In addition, LUNAR1 may be involved in conferring 5-FU resistance, being positively regulator of IGF-1R expression [188]. In human CRC cells, IGF-1R suppression could improve 5-FU-induced cell apoptosis and viability inhibition [210].

### Notch-associated lncRNAs in relation to metastasis or recurrence in CRC (Table 6)

**Table 6:** Notch-associated lncRNAs in relation to metastasis or recurrence in CRC

Notch-associated lncRNA	CRC				Refs.
	Notch regulated	miR sponged	Target protein	Recurrence ±	
UICLM	–	miR-215	ZEB2	Liver metastasis	[135]
DSCAM-AS1	–	miR-216b		Higher levels are associated with metastasis	[128, 213]
LINC00152	–	miR-185-3p and miR-632	-FSCN1 -E-cadherin & mesenchymal markers vimentin & N-cadherin through interacting with β-catenin	Promotes colon cancer cells invasion & metastasis	[215] [217]
LINC00707	Notch3	–	–	Recurrence	[130, 218]

UICLM increased level promotes liver metastasis in CRC through positively regulation of zinc finger E-box binding homeobox 2 (ZEB2) expression by sponging miR-215 [135]. ZEB2 is an E-cadherin transcriptional repressor regulates EMT [211, 212]. Additionally, expression of DSCAM-AS1 was significantly increased in patients with tumor metastasis compared to non-metastasis patients [128, 213]. DSCAM-AS1 induces invasion and migration of CRC cells through negative regulation of miR-216b [213].

Moreover, LINC00152 upregulation promotes CRC metastasis through positively regulation of Fascin actin-bundling protein 1 (FSCN1) expression via sponging miR-185-3p and miR-632. Fascin plays an important role in creating actin-based cellular protrusions, promoting motility and migration of CRC cells [214–216]. LINC00152 silencing in colon cancer cells was associated with increased E-cadherin level and decreased levels of mesenchymal markers vimentin and N-cadherin [217]. LINC00152 overexpression consistently contributed to epithelial properties loss and the development of mesenchymal traits in colon cells, promoting colon cancer cellular invasion and metastasis through interacting with β-catenin [217]. Beside, LINC00707 positively regulates Notch3 expression [130]. Notch3 increased nuclear expression has been attributed to tumor recurrence and could be used as a potential predictor in recurrent stages II and III CRC [218].

### Notch activation to facilitate CRC metastasis, mediated via EMT process

**Fig. 5:**
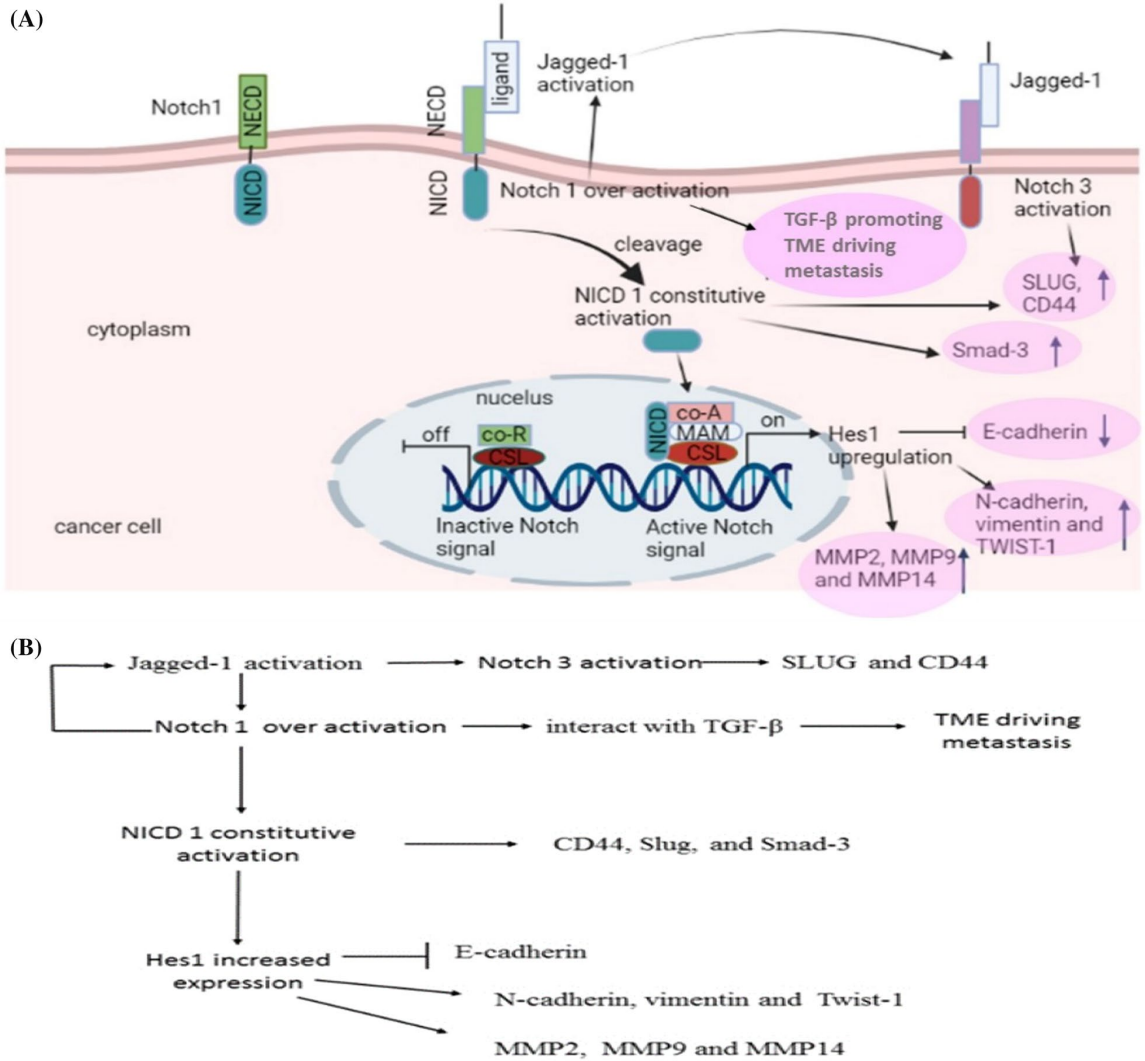
**A** Notch signaling induced EMT in CRC. *MMP* matrix metalloproteinases, *SLUG* snail family transcriptional repressor 2, Smad-3; SMAD family member 3, *TGF β* Transforming Growth Factor β.** B** Notch signaling induced EMT in CRC

EMT has contributed to a crosstalk between Notch receptors and their ligands in CRC [219] (Fig. 5A and 5B).

Prolonged Notch1 activation in the epithelial cells cause a senescence-like state, allowing tumor cells to trans-migrate from the main tumor and recruitment to distant locations [21]. Notch1 overexpression enhances Snail expression and inhibits E-cadherin expression in the immortalized endothelium cells, with induction of EMT and malignant transformation [220]. Activation of Notch-signaling contributes to hypoxia induced tumor cells invasion and migration [221].

In CRC, Notch1-signal enhances EMT, due to its interaction with transcription factor controlling EMT and TGF-β, promoting more TME driving metastasis [16]. Likewise, Fender et al., showed that higher level of the EMT-related proteins CD44, snail family transcriptional repressor 2 (SLUG), and SMAD family member 3(Smad-3), as well as phenotypic alterations in CRC cells, emerged from constitutive activation of NICD1 in CRC cells [22].

Activity of Jagged-1 is regulated by Notch1, which then activates Notch3, leading to an increased production of SLUG and CD44 [22].

Sonoshita et al., showed that inhibition of Notch-signal causes suppression in CRC tumor invasion and intravasation activated by knockdown of *Amino-terminal enhancer of split (Aes)* gene in *Apc*^Δ716^mice intestinal polyposis, pointing out to Notch-signal inhibition as a potential player during CRC metastasis prevention and treatment [222].

The Notch-related protein, Hes1, enhances CRC metastasis through induction of EMT; Upregulation of Hes1 contributed to loss of cell adhesion via repressing E-cadherin expression and enhancing N-cadherin, vimentin as well as the EMT inducer Twist-1 expressions [223]. Moreover, Hes1 overexpression was associated with an increased matrix metalloproteinases members (MMP2 and MMP9) mRNA levels in CRC cells, promoting tumor invasion [223]. Furthermore, Hes1 increases invasion via positively regulating MMP14 expression, mediated through STAT3 activity upregulation [224]. Therefore, patients with an increased Hes1 in stage II CRC, would have a higher recurrence rate chances after treatment [23].

### Conclusion and prospective

CRC has recognized as a dominant public health issue due to its high frequency and fatality rates [1]. Patients' prognosis remains poor despite substantial advancements in its treatments. Additionally, post-surgical relapse and metastases occur frequently [2, 4, 225]. Therefore, it is crucial to consider attentively the developments of novel biomarkers for CRC prognosis and treatment. Since the disruption of molecular pathways is a distinct characteristic of CRC, a variety of evaluations have suggested that pathways could be used as CRC treatment targets [226].

Recent studies have confirmed the major role of Notch signal in CRC progress. Notch signaling is capable of controlling both the homeostatic self-renewal and tumorigenic transformation of intestinal epithelial cells [15]. Additionally, epigenetic modifications have been shown to greatly contribute to occurrence and progression of inflammation enhanced CRC, and understanding of these alterations will aid to novel therapeutic alternatives detection [227]. The interplay of lncRNAs and Notch signal introduces innovative suggestion for CRC medication development.

In this review, we illustrated that Notch-associated lncRNAs displayed pivotal epigenetic regulatory role among cancer different aspects (growth, resistance, recurrence, and metastasis). The review summarized these regulatory control/involvement to come to a clearer understanding of Notch-related lncRNAs and their mechanisms upon cancer cells and the reverse, in CRC or other various cancer types. We enumerated a list of lncRNAs, described to influence, or are influenced by Notch-signaling activation, leading to colorectal tumorigenesis. Dysregulation of Notch-associated lncRNAs revealed to be highly associated with CRC progression/recurrence or conferring MDR as well as being involved in CRC metastasis. Thence, Notch-associated lncRNAs might be useful prognostic biomarkers or promising potential therapeutic targets for CRC treatment.

However, the impact of Notch associated lncRNAs in CRC, Few studies are available about lncRNAs GNAS-AS1 and RP11-89K10.1 and their impact on CRC clinical outcomes is still unknown, and their function(s) in CRC require further identification. The direct interaction between lnc34a and Notch-signaling is not fully elucidated. Additionally, other transcriptional regulators as histone modification, chromatin remodeling, and X chromosome inactivation to be addressed in another review.

## Data Availability

Not applicable.

## Abbreviations

ABC: ATP-binding cassette
ADAM: A disintegrin and metalloproteinase
Aes: Amino-terminal enhancer of split
APC: Adenomatous polyposis coli
AKT: Protein kinase B
Bcl-2: B-cell lymphoma-2
BRAF: V-raf murine sarcoma viral oncogene homolog B1
CD133: Prominin-1
ceRNA: Competitive endogenous RNA
CRC: Colorectal cancer
CSL: CBF1, Suppressor of Hairless, Lag-1
Co-A MAM: Co-A activators mastermind
CIN: Chromosomal instability
CIM: CpG island methylation
COX2: Cyclooxygenase-2
CXCL5: C-X-C Motif Chemokine Ligand 5
DCLK1: Doublecortin-like kinase 1
EMT: Epithelial-mesenchymal transition
eRNAs: Enhancer RNAs
ERBB4: Erb-b2 receptor tyrosine kinase 4
FSCN1: Fascin actin-bundling protein 1
5-FU: 5-Fluorouracil
Hes1: Hairy and enhancer of split-1
HCG11: 18; Human leucocyte antigen complex group 11, 18
HEY1: Hairy/enhancer-of-split related with YRPW motif protein1
HIF1α: Hypoxia-inducible factor 1α
IGF-1R: Insulin-like growth factor 1 receptor
IFN-γ: Interferon gamma
IL-1β: Interleukin-1β
Kras: Ki-ras2 Kirsten rat sarcoma viral oncogene homolog
KLF2: Kruppel-like factor 2
LncRNAs: Long non-coding RNAs
LINC00261: Long Intergenic Non-Protein Coding RNA 261
MAML: Mastermind like transcriptional coactivator
MATH1: A mouse homolog of the Drosophila proneural gene atonal
MDR: Multidrug resistance
MiRNAs: Micro-RNAs
MSI: Microsatellite instability
MMR: Mismatch repair genes
MMP: Matrix metalloproteinases
*MLH1*: *mutL homolog 1*
*MSH2*: *mutS homolog 2*
NATs: Natural antisense transcripts
NcRNAs: Non-protein coding RNAs
NECD: Notch extracellular domain
NICD: Notch intracellular domain
NSCLC: Non-small cell lung cancer
OS: Overall survival
PD-1: Programmed cell death protein-1
P-gp1: P-glycoprotein 1
PMS2: PMS1 homolog 2
RBPJ transcription factor: Recombination signal binding protein for immunoglobulin kappa J region
RNP: Ribonucleoprotein
SLUG: Snail family transcriptional repressor 2
Smad3: SMAD family member 3
SNHG3: 7, 12, Small nucleolar RNA host gene 3, 7, 12
Stat3: Signal transducer and activator of transcription 3
TFs: Transcription factors
TGF β: Transforming Growth Factor β
TNF-α: Tumor necrosis factor alpha
TNM: Tumor-lymph node-metastasis
TM4SF1: Transmembrane 4 L six family member 1
TP53 or p53: Tumor protein p53
VEGF: Vascular endothelial growth factor
YAP1: Yes-associated protein 1
YBX1: Y-Box Binding Protein 1
ZEB2: Zinc finger E-box binding homeobox 2
